# Cardiomyopathy and pregnancy

**DOI:** 10.1136/heartjnl-2018-313476

**Published:** 2019-07-15

**Authors:** Maria Schaufelberger

**Affiliations:** Molecular and Clinical Medicine, University of Gothenburg, Gothenburg, Sweden

**Keywords:** hypertrophic cardiomyopathy, idiopathic dilated cardiomyopathy, arrhythmogenic right ventricular dysplasia, pregnancy, heart failure

## Abstract

Cardiomyopathy is a group of disorders in which the heart muscle is structurally and functionally abnormal in the absence of other diseases that could cause observed myocardial abnormality. The most common cardiomyopathies are hypertrophic and dilated cardiomyopathy. Rare types are arrhythmogenic right ventricular, restrictive, Takotsubo and left ventricular non-compaction cardiomyopathies. This review of cardiomyopathies in pregnancy shows that peripartum cardiomyopathy is the most common cardiomyopathy in pregnancy. Peripartum cardiomyopathy develops most frequently in the month before or after partum, whereas dilated cardiomyopathy often is known already or develops in the second trimester. Mortality in peripartum cardiomyopathy varies from <2% to 50%. Few reports on dilated cardiomyopathy and pregnancy exist, with only a limited number of patients. Ventricular arrhythmias, heart failure, stroke and death are found in 39%–60% of high-risk patients. However, patients with modest left ventricular dysfunction and good functional class tolerated pregnancy well. Previous studies on >700 pregnancies in 500 women with hypertrophic cardiomyopathy showed that prognosis was generally good, even though three deaths were reported in high-risk patients. Complications include different types of supraventricular and ventricular arrhythmias, heart failure and ischaemic stroke. Recent studies on 200 pregnancies in 100 women with arrhythmogenic right ventricular cardiomyopathy have reported symptoms, including heart failure in 18%–33% of pregnancies. Ventricular tachycardia was found in 0%–33% of patients and syncope in one patient. Information on rare cardiomyopathies is sparse and only presented in case reports. Close monitoring by multidisciplinary teams in referral centres that counsel patients before conception and follow them throughout gestation is recommended.

## Introduction

Mothers are estimated to have any type of cardiovascular disease in 1%–4% of all pregnancies. This is the most common non-obstetric cause of maternal death.^1^ In the UK, maternal deaths from cardiovascular reasons accounted for 2.4/100 000 maternities in 2013–2015.^1^ Treatment of many cardiac diseases, including cardiomyopathy and care of the pregnant mother and fetus/child, has improved. Therefore, attitudes towards pregnancy have changed from caution in many women with cardiac disease to pregnancy being monitored by a joint multidisciplinary team, with specialists including obstetricians, cardiologists, anaesthesiologists and neonatologists.

## Cardiomyopathies

### Definition, classification and aetiology

**Figure 1:**
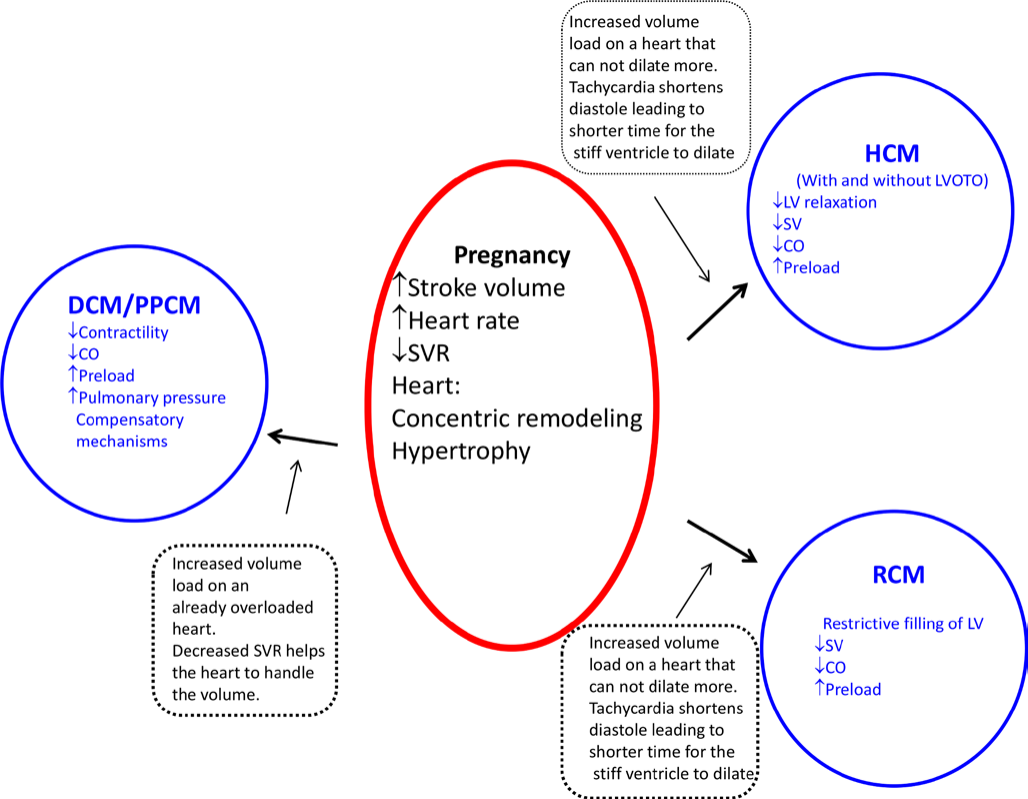
General description of haemodynamic changes during pregnancy and their effect on different types of cardiomyopathy. DCM, dilated cardiomyopathy; CO, cardiac output; HCM, hypertrophic cardiomyopathy; LV, left ventricle; PPCM, peripartum cardiomyopathy; SV, stroke volume; SVR, systemic vascular resistance.;RCM, restrictive cardiomyopathy; LVOTP, left ventricular outflow tract obstruction.

Cardiomyopathy is defined as a ‘myocardial disorder in which the heart muscle is structurally and functionally abnormal, in the absence of coronary artery disease, hypertension, valvular disease and congenital heart disease sufficient to cause the observed myocardial abnormality’^2^ (figure 1). Cardiomyopathies can be either acquired or inherited and include different types, such as hypertrophic cardiomyopathy (HCM), arrhythmogenic right ventricular cardiomyopathy (ARVC), left-ventricular non-compaction, restrictive forms (RCM) and dilated cardiomyopathy (DCM).^3^ DCM is a heterogenous group including idiopathic and inherited forms. DCM can be induced by viral infections, inflammatory diseases, tachycardia, storage diseases, toxic substances (alcohol, other drugs and medication) and Takotsubo cardiomyopathy. Peripartum cardiomyopathy (PPCM), which is the most common cardiomyopathy found in pregnancy, is often included in DCM.

Cardiomyopathies in pregnancy are generally not well described because they are relatively rare diseases. However, during the last decade, case series, case reports and a small number of controlled studies on cardiomyopathy in pregnancy have been published. In this review, different types of cardiomyopathies and pregnancy are described.

### Epidemiology

In the adult population, HCM and DCM have an estimated prevalence of 0.2%–0.4%. The estimated prevalence of ARVC is 0.02%–0.05% and restrictive cardiomyopathy is even more uncommon. HCM and ARVC predominantly have a genetic cause, while DCM and RCM have mixed causes.^3^ Even though HCM is the most common inherited cardiomyopathy, it has been identified in only 0.2% of deliveries.^4^

The Kaiser Permanente Health system, which is the USA’s largest non-profit health plan including 9.9 million members, provided data that identified pregnant women with heart failure from 2003 to 2014.^5^ Among these women, PPCM occurred in 333 (68.2%), non-ischaemic cardiomyopathy occurred in 34 (6.9%) and HCM occurred in 17 (3.5%) women.

The Registry Of Pregnancy And Cardiac disease (ROPAC) study, which was a voluntary registry for pregnancy and heart disease managed by the European Society of Cardiology Heart Survey Programme, included 1321 women with cardiac disease from 2007 to 2011. Of these, 89 patients had cardiomyopathy, 32 had DCM, 25 had PPCM, 27 had HCM and 5 had other cardiomyopathies.^6^ Patients with cardiomyopathy had the highest mortality rate (2.4%), as well as the highest incidence of arrhythmia and heart failure. A study from a French referral hospital reported 43 pregnancies in 36 women.^7^ In this study, 10 women had DCM, 28 had HCM, 3 had ARVC and 1 each had tachycardia-induced cardiomyopathy and left ventricular non-compaction cardiomyopathy.

### Haemodynamic changes during normal pregnancy

In normal pregnancy, cardiac output increases by 30%–50% through increased stroke volume during the first two trimesters. During the second part of pregnancy, cardiac output increases through an increase in heart rate of 10–15 beats/min (secondary to increased sympathetic tone); however, this increased rate does normally not reach >90 beats/min. The increase in plasma volume during pregnancy is larger than the increase in red blood cells, which leads to physiological anaemia. Systemic vascular resistance decreases at the end of the second trimester and then increases towards the end of pregnancy. The heart undergoes concentric remodelling and/or a mild eccentric hypertrophy.^8^ During labour, cardiac output increases progressively by as much as 80% directly after delivery. Blood loss during a normal delivery may be 500–1000 mL but is partly compensated by autotransfusion from the uterus during contractions and from the uteroplacental circulation after relief of vena caval compression by the uterus. Haemodynamic changes are fully reset after 6 months. During pregnancy and postpartum, patients remain in a hypercoagulable state.

### Dilated cardiomyopathy

There have been few reports of pregnant patients with DCM because the literature recommends against pregnancy if the ejection fraction is <30%. Idiopathic DCM accounts for approximately 50% of all cases and 35% are inherited.^3^ Other forms of DCM are rare, and information on pregnancy in patients with these forms is only found in case reports.

#### Diagnosis

##### Symptoms and signs

Either the diagnosis of DCM is already known or overt heart failure can develop secondary to asymptomatic left ventricular dysfunction and haemodynamic changes during pregnancy. Even if DCM is diagnosed before conception, symptoms can appear acutely or insidiously. Many symptoms of heart failure are compatible with those related to pregnancy; therefore, diagnosis of cardiomyopathy may be delayed.^9^ Recognising this condition and not misunderstanding the symptoms of heart failure (exertional dyspnoea, orthopnoea, leg oedema and fatigue) as secondary to pregnancy are important.^10^

##### Investigations

Biomarkers, such as brain natriuretic peptide/N-terminal probrain natriuretic peptide, support a diagnosis of heart failure. Echocardiography shows the current status of the heart. Because gadolinium contrast crosses the placenta, late gadolinium MRI should be avoided during pregnancy.

#### Adverse events

Because of increased haemodynamic load and cessation of heart failure medication, adverse cardiac events in women with cardiac disease are more likely to appear towards the end of pregnancy. This is especially the case in higher New York Heart Association functional classes and in moderate to severe left ventricular dysfunction where adverse cardiac events are reported in 39% and 60% of pregnancies, respectively.^10 11^ Adverse events include heart failure and/or ventricular tachycardia, aborted sudden death, atrial fibrillation, transitory ischaemic attack and death. In one study that included 32 pregnant women (16 with idiopathic DCM and 5 with doxorubicin-induced DCM), 14 had a cardiac event.^10^Heart failure occurred in nine women in this study, arrhythmia in seven women and one woman had a transient ischaemic event/stroke. This led to a 16-month event-free survival of 28% in pregnant women with DCM versus 83% in non-pregnant women with DCM. In 10 pregnancies in another study, six patients had a major adverse event and two patients died from cardiovascular complications; these patients chose not to follow a multidisciplinary management plan (table 1).^11^ Tachycardia-induced DCM is often treatable, and many patients improve or even recover a normal ejection fraction.^12^ One case report included a patient with an earlier episode of atrial flutter and heart failure.^8^. She developed atrial fibrillation and heart failure, which required intensive care during pregnancy. This patient was treated with radiofrequency ablation.

**Table 1:** Pregnancy in dilated cardiomyopathy, complications, mortality and treatment

Author	Year	Women	Pregnancies	Arrhythmia	HF	CVA	CS	Abortion	Deaths	Diuretics	ACE-I	Digoxin	BB	Anti-arrhythmic	ICD
Siu *et al* 11	2001	NA	23	4 (17) SVT	7 (30)	1 (4)	NA	NA	1 (4)	NA	NA	NA	NA	NA	NA
Grewal *et al* 10	2010	32	36	1 (3) VT 5 (16) AF	9 (28)	1 (3)	7 (19) obstetric reason	1 (3) therapeutic	0	3 (9)	2 (6)	7 (22)	6 (19)	NA	1 (3)
Roos-Hesselink *et al* 6	2013	NA	32	NA	NA	NA	NA	NA	1 (3)	NA	NA	NA	NA	NA	NA
Avila *et al* 43	2003	27	27	NA	NA	2 (7)	NA	NA	3 (11)	NA	NA	NA	NA	NA	NA
Billebeau *et al* 7	2018	9	10	1 (11) AF 1 (11) aborted SD	3 (33)	2 (22)	NA	NA	2 (22)	NA	NA	NA	NA	NA	NA

All values are n (%).

ACE-I, ACE inhibitor; AF, atrial fibrillation; BB, beta-blocker; CS, caesarean section; CVA, cardiovascular accident; HF, heart failure; ICD, implantable cardioverter defibrillator; NA, not available; SD, sudden death; SVT, supraventricular tachycardia; VT, ventricular tachycardia.

A previous cardiac event is the most predictive risk factor for a subsequent major event.^8^ However, women with no history of cardiac events, a good New York Heart Association class and only a slight effect on the left ventricle have a good chance of a pregnancy free from major events.^10^ With improved treatment of DCM and underlying diseases, the ejection fraction may recover in some patients.

#### Peripartum cardiomyopathy

##### Definition

A commonly used definition of PPCM is symptoms of heart failure secondary to left ventricular systolic dysfunction with an ejection fraction <45% towards the end of pregnancy or in the months following delivery with no other cause of heart failure.^13^ Most cases develop around childbirth.

##### Epidemiology

The reported incidence of PPCM varies between 1:100 and 1:20 000 deliveries worldwide and between races within countries, with the highest incidence in African countries and the lowest in Scandinavia and Japan. An increasing trend of PPCM has been reported and is likely secondary to greater knowledge about and attention to this disease.^14^ The mortality rate varies from <2%^14^ to 50%.^15^

##### Predisposing factors

Known predisposing factors of PPCM are multiparity, African ethnicity, malnutrition, increasing age and young age, pre-eclampsia and traditional risk factors for cardiovascular disease, such as diabetes and smoking.

##### Pathophysiology

The most probable aetiology of PPCM is a multifactorial aetiology, including inflammation, angiogenic imbalance and genetic factors inducing apoptosis and vascular damage.^16^ Right ventricular involvement, accompanied by less recovery of the left ventricle, was observed in 60% of patients in one study.^17^

The EURObservational Research Programme (EORP) has completed enrolment of 766 patients with PPCM worldwide. This registry provides information on risk factors, symptoms, clinical findings, investigations, treatment, repeated pregnancies and prognoses. Because PPCM is a diagnosis of exclusion, obtaining information about risk factors and other medical conditions is important, and typical signs of heart failure are most often present. In 411 patients with PPCM who were included in the EORP registry, 6.8% developed some type of thromboembolic event.^18^

#### Management of DCM and PPCM

In DCM, specific treatment against the aetiology may be possible. Moreover, disease management is common for most DCM subtypes and PPCM. Symptoms of DCM or PPCM can develop before and after parturition. Because these symptoms can be mild or life threatening, the patient’s general status must be assessed first.

##### Acute, prepartum and postpartum treatment

**Figure 2:**
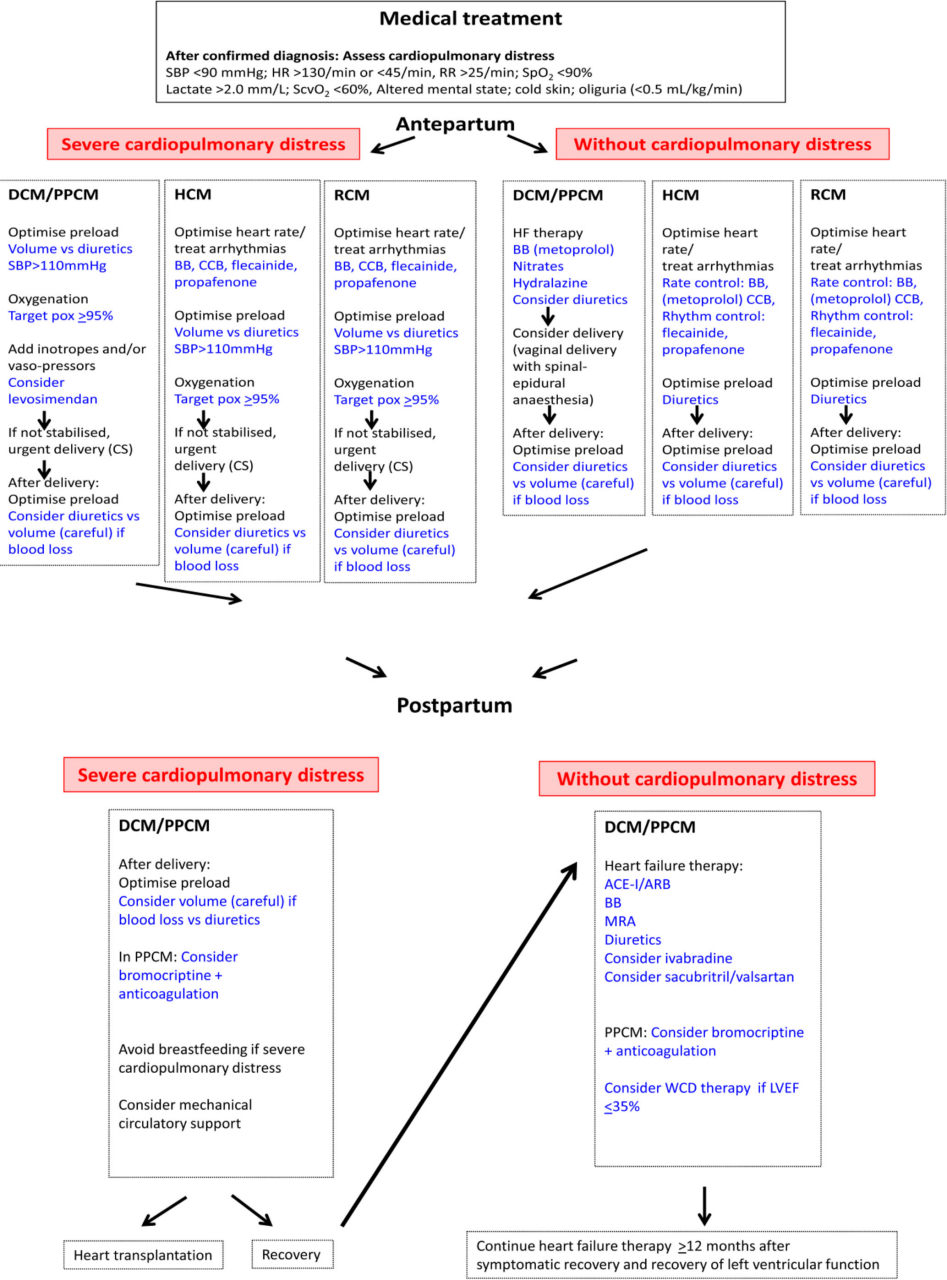
Treatment algorithm for DCM/PPCM, HCM and RCM during pregnancy and after delivery. ACE-I/ARB; ACE inhibitor/angiotensin receptor blocker; BB, beta-blocker; CCB, calcium channel blocker; CS, caesarean section; DCM, dilated cardiomyopathy; HCM, hypertrophic cardiomyopathy; HR, heart rate; MRA, mineral corticoid receptor antagonist; PPCM, peripartum cardiomyopathy; RR, respiratory rate; SBP, systolic blood pressure; ScvO_2_, central venous oxygen saturation; SpO_2_, peripheral oxygen saturation; WCD, wearable cardioverter defibrillator.

If the patient is pregnant, the first decision is whether she can complete pregnancy or if it must be interrupted. If pregnancy is continued, treatment must be adjusted while considering the fetus.^19^ Table 2 summarises treatment that is recommended for DCM and PPCM. Figure 2 summarises medical treatment for all cardiomyopaties.

**Table 2:** Summary of cardiac medication during and after pregnancy in dilated cardiomyopathy and peripartum cardiomyopathy

Drug	Pregnancy	Recommendation during pregnancy	Recommendation during breastfeeding	Postpartum
ACE-I/ARB	Teratogenic. 48% and 87% of fetuses exposed to ACE-I and ARB, respectively, had some type of complication.	Contraindicated.^22^	Captopril, benazepril and enalapril are considered safe.^22^ *Data are limited for other preparation* 22	Used according to guidelines.^26^
ARNI	See ACE-I/ARB.	Contraindicated.^22^	Limited data.^22^	One case report with positive effect in PPCM.
Beta-blockers	Shall be used. Can give babies hypoglycaemia, bradycardia and small for gestational age. Metoprolol is recommended.^22^	Metoprolol and carvedilol are considered safe.^22^ Atenolol is contraindicated.^22^	Metoprolol: acceptable.^22^ Carvedilol: unknown.^22^	Used according to guidelines.^26^
MRA	Spironolactone have antiandrogenic effects.^22^ Eplerenone in high doses have caused postimplantation losses in rabbits.^22^	Contraindicated.^22^	Not recommended.^22^	Used according to guidelines.^26^
Diuretics	Oligohydramnios and electrolyte disturbances and insufficient human data.^38^	Furosemide and bumetanide are considered safe.^22^	Furosemide, limited data, acceptable.^22^	Used according to guidelines.^26^
Inotropes	Levosimendan is recommended in PPCM even if human data are inadequate.^26^	Levosimendan may be preferred inotrope.^26^	Unknown.^22^	Used according to guidelines.^26^
Vasodilators	Hydralazine and high-dose long-acting nitrates are recommended.^26^ Hydralazine is teratogenic in mice.	Conflicting data.	Hydralazine: considered safe.^22^ Isosorbide dinitrate: unknown.^22^	Change to ACE-I/ARB/ARNI.
Ivabradine	Teratogenic in animals.^22^	Do not use.	Unknown.^22^	Positive effects in PPCM.
Anticoagulation	LMWH if needed. Interrupted 4–6 hours before planned delivery.	Considered safe.^26^	Considered safe.^26^	Continued 4–6 weeks postpartum^34^ and used according to guidelines.^26^
Digoxin	Placenta permeable. Safe.^38^	Considered safe.^22^	Minimal exposure.^22^	Used according to guidelines.^26^
Bromocriptine	–			2.5 mg ×1 in 1 week in mild PPCM, 2.5 mg ×2 in 2 weeks and 2.5 mg ×1 in 6 weeks if EF <25% or cardiogenic chock, combined with anticoagulation.^26^

ACE-I, ACE inhibitor; ARB, angiotensin receptor blocker; ARNI, angiotensin receptor neprilysin inhibitor; EF, ejection fraction; LWMH, low weight molecular heparin; MRA, mineral corticoid receptor antagonist; PPCM, peripartum cardiomyopathy.

##### Chronic phase

Treatment for heart failure should follow the 2016 European Society of Cardiology Guidelines for the treatment of acute and chronic heart failure, as well as the 2017 ACC/AHA/HFSA Focused Update and the 2013 ACCF/AHA Guideline for the Management of Heart Failure.

##### Bromocriptine

Bromocriptine blocks prolactin. In a small, randomised study, bromocriptine hastened recovery of the ejection fraction compared with conventional treatment.^20^ In 63 patients who were randomised to low-dose bromocriptine or high-dose bromocriptine for 2 weeks followed by low-dose therapy for 6 weeks, both groups showed a marked increase in left ventricular function compared with results reported in the Investigations of Pregnancy-Associated Cardiomyopathy study.^21 22^ Longer treatment tended to result in full recovery^21^ and is recommended in patients with an ejection fraction of <25% or in patients with cardiogenic shock.^23^ In uncomplicated cases, low-dose treatment can be considered.^23^ Bromocriptine should be combined with at least a prophylactic dose of unfractionated heparin or low-molecular-weight heparin^24^ because rare case reports have described cardiac complications of coronary thrombosis with bromocriptine.

##### Wearable cardioverter defibrillator

Careful discussion is necessary before installing an implantable cardioverter defibrillator because PPCM often improves. However, particularly initially, there is a high risk of malignant arrhythmia in PPCM. This is why a wearable cardioverter defibrillator is recommended in patients with an ejection fraction ≤35% for the first 3–6 months.^25^ Thereafter, a decision about installing an implantable cardioverter defibrillator must be made.

##### Length of treatment for patients with PPCM

Treatment is recommended for at least 12 months after recovery of both the left ventricular ejection fraction and dimensions.^24^ In our clinic, we continue treatment until patients have been asymptomatic and maintained an ejection fraction ≤50% for 12 months before starting to taper mineralocorticoid receptor antagonists, followed by the lesser tolerated ACE inhibitors and beta-blockers. To date, we have stopped medication in 63% of patients without any relapse.

##### Delivery and breast feeding

If a patient cannot be stabilised haemodynamically, urgent delivery by caesarean section is necessary. Vaginal delivery is preferred in haemodynamically stable patients.^23^ Breast feeding is discouraged in patients with New York Heart Association class III–IV because of its high metabolic demand.

##### Subsequent pregnancies, counselling and contraception

Strong risk factors for deterioration of left ventricular function and death are an ejection fraction <20%, right ventricular failure, mitral regurgitation, atrial fibrillation and/or hypotension.^23^ A joint multidisciplinary team is indicated for counselling and during pregnancy. Many heart failure medications are contraindicated during pregnancy (table 2).^19^ Therefore, changing medications, such as blockers of the renin–angiotensin–aldosterone system, to hydralazine and long-acting nitrates is advisable. Beta-blockers should be continued. During medication changes and pregnancy, careful follow-up of patients’ signs and symptoms, monitoring of brain natriuretic peptide/N-terminal probrain natriuretic peptide levels and repeated echocardiography are necessary. Changes to a patient’s risk profile indicate the need for additional counselling.

Information on subsequent pregnancies in patients with PPCM is limited, which has led to high variation in reports. Each patient should have the option to meet with a joint multidisciplinary team to discuss the risk of PPCM relapse. The rate of PPCM relapse is 14%–44% if the ejection fraction has recovered and 16%–56% if the ejection fraction has not normalised. Mortality in subsequent pregnancies in PPCM vary (0%–48%)%).^26–28^ Very high mortality has been reported, especially if the ejection fraction was not normalised. In that case, patients with PPCM should be advised against pregnancy. A period of time without medication and with recurrent echocardiograms is ideal for confirming that the ejection fraction does not deteriorate before a decision regarding a new pregnancy.

Contraception must be discussed in all patients with DCM and PPCM, preferably before discharge. Progesterone-only devices or pills are recommended.

#### Hypertrophic cardiomyopathy

Hypertrophy can be general or local and is often located in the left ventricular outflow tract, which may lead to outflow tract obstruction. The diagnosis of HCM is made with echocardiography.

The increase in plasma volume during pregnancy may reduce the left ventricular outflow gradient. However, arrhythmia and hypovolaemia may cause an increase in the left ventricular outflow gradient in patients with obstruction of the outflow tract. Generally, women with HCM tolerate pregnancy well, even though symptoms, such as dyspnoea, heart failure, arrhythmia, angina, dizziness and syncope, occur in up to 48% of affected patients, especially in patients with symptoms before pregnancy (table 3).^6 7 29 30^ Patients with HCM have an increased risk if they are symptomatic, they have a history of arrhythmia or they have significant left ventricular outflow tract obstruction, diastolic dysfunction,^29 31^ a CARPREG or ZAHARA score ≥1 (scoring systems in congenital heart disease to predict the risk of cardiac complications) or medication before pregnancy.^32^ However, recent reports from the ROPAC registry and Billebeau and coworkers showed no difference in outcome between patients with and those without outflow obstruction,^7 30^ but symptoms and signs of heart failure before pregnancy were associated with a major event.^30^ In a meta-analysis of 237 women and 408 pregnancies, the mortality rate of HCM was 0.5%.^33^ In an Italian study, the maternal relative mortality risk in HCM compared with the expected risk was 17.1 (two observed deaths, where 0.12 would have been expected, but with wide CIs (2.0 to 61.8)).^29^

**Table 3:** Pregnancy in hypertrophic cardiomyopathy: symptoms, complications, delivery and mortality

Author	Year	Women	Pregnancies	Arrhythmia	Syncope	HF	CVA	Chest pain	CS	Abortion	Deaths
Turner *et al* 44	1968	9	13	2 (22) tachycardia.	1 (11)	5 (56) dyspnoea. 2 (22) HF.	NA	2 (22)	4 (44)	3 (33)	0
Oakley *et al* 45	1979	23	54	NA	NA	10 (43) dyspnoea.		2 (9)	10 (43)	1 (1.8) medical. 10 (18) spontaneous.	0
Siu *et al* 11	2001	9	NA	1 VT	NA	NA	NA	NA	NA	NA	0
Autore *et al* 29	2002	100	199	1/40 (2.5) AF.	1/40, (2.5) during labour	6/40 (15) dyspnoea/HF.	NA	NA	15/40 (38)	NA	2/100 (2)
Thaman *et al* 46	2003	127	271	9 (7) palpitations.	2 (1.6)	6 (5) dyspnoea. 2 (1.6) HF postpartum.	NA	12 (9)	19 (15)	NA	0
Avila *et al* 43	2003	15	15	3 (2) AF+1 NA.	0	5 (33)	0	5 (33)	NA	NA	0
Walker *et al* 47	2007	10	11	NA	NA	NA	NA	NA	2 (20) obstetric.	0	0
Avila *et al* 48	2007	23	23	2 (9) AF. 1 (4) SVT.	NA	7 (30)	1 (4)	NA	12 (52) obstetric.	NA	0
Schuler *et al* 49	2012	8	12	1 (12) SVT. 1 (12) VT-antitachycardia pacing. 1 (12) non-sustained VT.	NA	2 (24)	1 (12)	NA	4 (50)	1 (12) foetal reason.	0
Sikka *et al* 4	2014	4	4	3 (75) palpitations.	NA	1 (25) HF. 3 (75) dyspnoea.		1 (25)	0	0	0
Tanaka *et al* 32	2014	23	27	1 (4) VT 6 (26) non-sustained VT. 6 (26) PVC.	NA	2 (8) worsening NYHA class.	NA	NA	9 (33)	4 (15) pregnancies terminated, cardiac reasons.	0
Ashikhmina *et al* 50	2015	14	23	6 (43) palpitations.	5 (36) syncope. 2 (14) presyncope.	7 (50) dyspnoea. 3 (21) HF postpartum.		2 (14)	11 (79)	NA	0
Lima *et al* 51	2015	52	NA	NA	NA	7 (13)	0	NA	25 (48)	NA	0
Goland *et al* 30	2017	60	60	6 (10) VT. 1 (17) AF.	NA	9 (15)	0	NA	36 (60)	0	0
Billebau *et al* 7	2018	22	28	1 (4) VT.	NA	3 (14)	NA	NA	2 (7) cardiac.	NA	1 (4)

All values are n (%).

AF, atrial fibrillation; CS, caesarean section; CVA, cardiovascular accident; HF, heart failure; NA, not available; NYHA, New York Heart Association functional class; PVC, premature ventricular contraction; SVT, supraventricular tachycardia; VT, ventricular tachycardia.

Evaluation of risk factors and counselling before conception is important. During pregnancy, moderate risk to high-risk patients should be followed each trimester if they are in WHO class II and followed monthly or every second month if in WHO class III. This should be performed to detect symptoms and signs and to perform echocardiography, especially following a left ventricular outflow tract gradient and monitoring heart rhythm. If a patient is already receiving a beta-blocker, this should be continued, and if more symptoms appear in beta-blocker-naive patients, a beta-blocker should be started (table 4, figure 2).^23^ Verapamil is an alternative if beta-blockers are not tolerated.^23^ If arrhythmia occurs, anticoagulation is recommended, and cardioversion is an option for poorly tolerated atrial fibrillation. Implantation of an implantable cardioverter defibrillator can be considered if indicated.^23^

**Table 4:** Treatment during pregnancy in hypertrophic cardiomyopathy

Author	Year	Women	Pregnancies	Diuretics	BB or CCB	Antiarrhythmic	Pacemaker	ICD
Turner *et al* 44	1968	9	13	2 (22)	8 (89)	0	NA	NA
Oakley *et al* 45	1979	23	54	6 (26)	18 (78)	NA	NA	NA
Siu *et al* 11	2001	9	NA	1 (11)	4 (44)	NA	NA	NA
Autore *et al* 29	2002	100	199	NA	10/40 (25) BB. 2/40 (5) CCB.	1/40 (2.5) amiodarone.	NA	NA
Thaman *et al* 46	2003	127	271	NA	15 (12) BB.	3 (2)	2 (1.5)	0
Avila *et al* 43	2003	15	15	NA	NA	NA	NA	NA
Walker *et al* 47	2007	10	11	3 (27)	NA	0	NA	5 (45)
Avila *et al* 48	2007	23	23	6 (26)	12 (52) BB and/or CCB.	NA	NA	NA
Schuler *et al* 49	2012	8	12	5 (62)	6 (75)	NA	NA	8 (100)
Sikka *et al* 4	2014	4	4	NA	1 (25)	0	0	0
Tanaka *et al* 32	2014	23	27	NA	12 (52) BB.	NA	NA	NA
Ashikhmina *et al* 50	2015	14	23	NA	13 (93) BB. 2 (14) CCB.	NA	NA	7 (50)
Lima *et al* 51	2015	52	NA	NA	NA	NA	NA	NA
Goland *et al* 30	2017	60	60	4 (7)	24 (4) BB. 5 (8) CCB.	3 (5)	NA	4 (7) 1 ICD direct postpartum.
Billebau *et al* 7	2018	22	28	0	15 (68)	1 (4.5) flecainide.	1 (4.5)	3 (14)

All values are n (%).

BB, beta-blocker; CCB, calcium channel blocker; ICD, implantable cardioverter defibrillator; NA, not available.

#### Delivery

Hypovolaemia secondary to blood loss and vasodilation is poorly tolerated. Addressing these issues and carefully choosing anaesthesia are important. Vaginal delivery with regional anaesthesia is preferred, but a plan for caesarean section, if necessary, should be prepared in high-risk patients. However, in the ROPAC study findings, only 5% of patients required emergency caesarean section.^30^ Overall recommendations discourage general anaesthesia because of the higher risk of complications.

## Other cardiomyopathies

### Left ventricular non-compaction

Left ventricular non-compaction is partially an inherited disease, which is characterised by hypertrabeculation with deep clefts in the myocardium and an increased risk of thromboembolic events.^3^ In 25% of normal pregnancies, there is a transient increase in left ventricular trabeculation,^34^ which makes diagnosis of left ventricular non-compaction during pregnancy more difficult. Left ventricular non-compaction in pregnancy has only been described in case reports. Pregnancy in left ventricular non-compaction is often complicated by heart failure and arrhythmias, but no mortality has been reported.^35 36^ There is no specific treatment, but anticoagulation is recommended for patients with a history of thromboembolic events, atrial fibrillation, intracardiac thrombi or impaired left ventricular function.

### Restrictive cardiomyopathy

Restrictive cardiomyopathy, which is a partly acquired and partly inherited disease, may affect both ventricles. Restrictive cardiomyopathy is complicated by increased myocardial stiffness and reduced relaxation that result in reduced ventricular filling. An increase in plasma volume during pregnancy may lead to volume overload and left or right heart failure. A case report described a pregnant patient with restrictive cardiomyopathy who was symptomatic before pregnancy.^37^ Another patient who developed supraventricular tachycardia and symptoms of heart failure during pregnancy was referred to our hospital. Echocardiography showed a restrictive filling pattern and normal ejection fraction. The patient in this case report and our patient were treated with beta-blockers and diuretics (figure 2) and successfully delivered vaginally.

### Arrhythmogenic right ventricular cardiomyopathy

ARVC is an inherited cardiomyopathy. Loss of cardiomyocytes is followed by fibrofatty replacement in the right ventricle that causes arrhythmia, as well as right heart failure. Occasionally, the left ventricle may also be involved. Increased plasma volume during pregnancy may provoke these complications. Recent studies that included approximately 200 pregnancies in 100 women with ARVC reported no mortality in relation to pregnancy (table 5).^7 38–41^ Women were symptomatic in 18%–33% of pregnancies with dizziness, dyspnoea, palpitations, heart failure, occurrence of ventricular tachycardia (0%–33%) and syncope. The large difference in reported ventricular tachycardia is most probably due to few patients in some reports. Many women were treated with an implantable cardioverter defibrillator before pregnancy. The most common antiarrhythmic drug, except for beta-blockers, was flecainide. The majority of women delivered vaginally without any complications. In ARVC, as in other inherited diseases, appropriate counselling is required before pregnancy. Symptomatic patients are advised not to become pregnant.^3^

**Table 5:** Pregnancy in arrhythmogenic right ventricular cardiomyopathy

Author	Year	Women	Pregnancy	Palpitation	Syncope/dizziness	Arrhythmia	HF	CS	Deaths	Diuretic	BB	Antiarrhythmic	ICD
Bauce *et al* 40	2006	6	6	2 (33)	NA	2 (33) VES. 1 (17) VT postpartum.	NA	4 (67)	0	NA	2 (33)	2 (33) propafenone. 2 (33) flecainide.	1 (17)
Hodes *et al* 41	2016	26	39	7 (27)	NA	5 (19) SVA.	2 (8)	11 (28)	0	3 (12)	16 (62)	1 (4) flecainide. 4 (15) sotalol.	28 (72) deliveries.
Castrini *et al* 38	2018	58	88	6 (10)	15 (26) syncope. 1 (2) dizziness.	22 (38) VA.	0	6 (7)	0	NA	5 (9)	NA	16 (28)
Gandjbakhch *et al* 39	2018	23	60, 50 completed	9 (39)	2 (9) dizziness.	1 (4) VT. 1 (4) undocumented tachycardia.	0	8 (16)	0	0	6 (26) BB only	2 (8) flecainide only. 5 (22) BB+flecainide.	Four during follow-up.
Billebeau *et al* 7	2018	3	3	NA	NA	1 (33) VT.	0	NA	0	NA	NA	1 (33) flecainide+catheter ablation.	NA

All values are n (%).

BB, beta-blocker; CS, caesarean section; HF, heart failure; ICD, implantable cardioverter defibrillator; NA, not available; SVA, sustained ventricular tachycardia, aborted sudden cardiac death or appropriate implantable cardioverter defibrillator (ICD) intervention; VA, ventricular arrhythmia; VES, ventricular extra systole; VT, ventricular tachycardia.

### Takotsubo cardiomyopathy

Most of the reported Takotsubo cardiomyopathy cases became symptomatic close to childbirth with caesarean delivery. One-third of these cases were found during caesarean section. All patients with Takotsubo cardiomyopathy recovered between 4 days and 3 months, as in non-pregnant patients with Takotsubo cardiomyopathy.^42^

## Conclusion

The most common complications during pregnancy in cardiomyopathy are heart failure and arrhythmia. Currently, pregnant patients with cardiomyopathy who were previously discouraged can, in many  cases, proceed with help from a multidisciplinary team, even if there is an increased risk for complications, especially in DCM. Therefore, these parents require proper counselling before conception and must be aware of the risks.
